# Fatigue Crack Growth Performance of Q370qENH Weathering Bridge Steel and Butt Welds

**DOI:** 10.3390/ma17236015

**Published:** 2024-12-09

**Authors:** Yujie Yu, Xiang Zhang, Chunjian Hu, Liangkun Liu, Haibo Wang

**Affiliations:** 1 National Engineering Research Center of High-Speed Railway Construction Technology, Central South University, Changsha 410075, China; 2 School of Civil Engineering, Central South University, Changsha 410075, China; zx13981668559@163.com (X.Z.); huchunjian0302@163.com (C.H.); 3 Key Laboratory of Intelligent Disaster Prevention and Emergency Technologies for Urban Lifeline Engineering, Dongguan 523808, China; 4 Hunan Tieyuan Civil Engineering Testing Co., Ltd., Changsha 410075, China

**Keywords:** Q370qENH weathering steel, fatigue crack growth, butt weld, heat-affected zone (HAZ), stress ratio, fracture morphology

## Abstract

Weathering steel possesses good atmospheric corrosion resistance and is increasingly applied in highway and railway bridges. The fatigue performance of the weld joint is an important issue in bridge engineering. This study experimentally investigates the microstructural properties and fracture crack growth behaviors of a Q370qENH bridge weathering steel weld joint. The FCG parameters of the base steel, butt weld, and HAZs, considering the effect of different plate thicknesses and stress ratios, are analyzed. Microstructural features, microhardness, and fatigue fracture surfaces are carefully inspected. The FCG rates of different weld regions in the stable crack growth stage are obtained using integral formulas based on the Paris and Walker law. The test results indicate that the heating and cooling process during the welding of Q370qENH steel creates improved microstructures with refined grain sizes and fewer impurities, thus leading to improved FCG performances in the HAZ and weld regions. The crack growth rate of Q370qENH weld regions increases with the stress ratio, and the influencing extent increasingly ranks as the base steel, HAZ, and the weld. The thick plate has a slightly slower fatigue crack growth rate for the Q370qENH weld joints. The Q370qENH base steel presents the highest fatigue crack growth rate, followed by the heat-treated and HAZ cases, while the weld area exhibits the lowest FCG rate. The Paris law coefficients of different regions of Q370qENH welds are presented. The collected data serve as a valuable reference for future analyses of fatigue crack propagation problems of Q370qENH steel bridge joints.

## 1. Introduction

Steel truss bridges possess many advantages such as low self-weight, strong span capacity, high prefabrication quality, and a short construction period, making them widely used in highway and railway bridge construction [1,2]. Corrosion resistance and fatigue life under vehicle load are critical issues in the design and maintenance of steel truss bridges [3,4]. Steel bridges often face rust issues during their actual operation. Traditionally coated bridges need regular recoating for corrosion prevention, which leads to high maintenance costs. There also exist difficulties in maintaining certain hidden regions of the bridge, along with pollution to the surrounding environment. Weathering steel is an improved steel product that possesses good atmospheric corrosion resistance [5,6]. By incorporating corrosion-resistant alloy elements such as Cu, P, Cr, and Ni, weathering steel promotes the formation of dense and stable rust layers on the metal surface, effectively preventing corrosive substances like moisture, CO_2_, and SO_2_ from reaching the base metal and causing corrosion [7]. Weathering steel has been applied in highway bridges in China for decades, and it has been implemented in railway bridges in recent years, such as the Zangmu Bridge and the Weilai Railway Bridges [8,9].

Many highway and railway weathering steel bridges utilize uncoated weathering steel and welded joints [10,11]. These structures are subjected to dynamic forces from vehicular traffic, trains, and wind-induced vibrations, potentially leading to fatigue fractures at the welded connections of decks and structural members. The fatigue performance of weathering steel and welded joints is an important concern during the application design and lifetime safety evaluation of weathering steel bridges. The generally applied approach for fatigue performance assessment is the fatigue test to determine the S-N (stress–fatigue life) relations. Su et al. [12,13,14] performed fatigue tests of Q345qNH butt-welded and filet-welded joints with different stress ranges to determine the S-N curves. Notably, fatigue cracks consistently manifested at the interface between the weld and base metal.

Zhao et al. [15] conducted fatigue tests on Q370qNH butt-welded, non-load-carrying T-shaped, and cruciform-welded joints to determine their failure modes and fatigue properties. Different S-N curve tangents were compared to the fatigue codes. The obtained S-N curves can be incorporated into a structural analysis to determine fatigue damage using the Palmgren–Miner rule and the rain-flow counting method [16]. Zhang et al. [17] and Gao et al. [18] investigated the fatigue properties of the base metal, butt-welded joints, and cruciform-welded joints of Q345NH and Q690qENH weathering steel, respectively. The results all indicated that welding material-matching and the welding process have a great impact on the fatigue properties. The S-N curve method offers a convenient way for fatigue safety evaluations, but the obtained fatigue properties represent the overall performance of the analyzed specimen and may not accurately indicate the specific damage conditions at particular locations [19]. Moreover, these properties often possess a high level of uncertainty.

The fracture-based fracture crack growth (FCG) method is another generally used approach for fatigue life evaluation, which is applied to evaluate the fatigue life with initial cracks [20]. Basic fatigue crack growth properties can be obtained through compact tensile tests, and then these properties can be incorporated to investigate the fatigue safety of complex steel bridge joints and the influence of corrosion pits or crack defects [21,22,23]. Ke et al. [24] investigated the FCG rate of WNQ570, Q370qE, and Q345qC, respectively, and found an increased crack growth rate with increased stress ratios. In terms of weathering steel, Su et al. [25] conducted CT tests on Q345 steel and butt welds. The results indicate higher fatigue crack growth thresholds than Q345qD steel. Tan et al. [26] performed the FCG test on Q690 high-strength steel. The results indicated that the Q690 weld presented a lower crack propagation rate at a low stress amplitude, but a higher crack propagation rate under cases with a high stress amplitude, compared to the base steel. The comparisons in these tests mainly feature the differences between welds and base steel. Discussions on the FCG performance of heat-affected zones (HAZs) are limited, even though the fatigue tests of welded joints often report crack initiation and propagation from the weld root or HAZs.

Q345qNH and Q370qNH are generally applied weathering steel grades. Many references can be found featuring fatigue test data on different grades of Q345q steel [13,14,24,25], but limited work has been reported on Q370qNH. This study aimed to experimentally investigate the microstructural characteristics and fracture crack growth (FCG) behavior of Q370qENH bridge weathering steel, particularly focusing on butt weld joints. The FCG parameters of base steel, butt weld, and HAZs, considering the effect of different plate thicknesses and stress ratios, were analyzed. The microstructural features of different regions of the weld joints were inspected using an optical microscope (OM) and an image processing system. A scanning electron microscope was used to observe the micromorphology of the fatigue fracture. Additionally, the FCG rates of different weld regions in the stable crack growth stage were obtained using integral formulas based on the Paris and Walker law. The tested value of Q370qENH was later compared with other grades of weathering steel reported in references and codes. The collected data serve as a valuable reference for future analyses of fatigue crack propagation problems.

## 2. Specimen Preparation and FCG Experimental Details

### 2.1. Material Properties

The bridge weathering steel investigated in this study, denoted as Q370qENH, is manufactured by Angang Steel Co., Ltd. (Anshan, China). Q370 signifies the nominal yield strength of 370 MPa at room temperature. The index *q* stands for bridge steel; *E* indicates the quality grade with at least 27J Charpy impact energy at −40 °C; and NH implies the weathering steel. To ensure the optimal atmospheric corrosion resistance for both the base steel and weld regions, the specifically matched soldering flux SJ105Q and submerged arc welding wire TH550-NQ-III are employed. The chemical compositions of the base steel Q370qENH, the flux, and the welding wire, along with the corresponding requirements outlined in Chinese standard GB/T 714-2015 [27], are provided in Table 1, Table 2 and Table 3. Mechanical properties are tested based on ISO 6892-1 [28], and the obtained properties are presented in Table 4.

**Figure 1:**
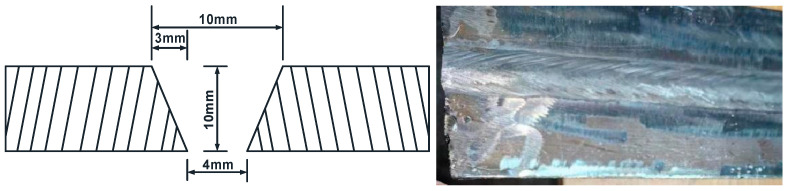
Design geometry and the practical product of butt welds.

The weld specimens are fabricated from 10 mm thick plates with the butt weld design illustrated in Figure 1. The butt weld joints are fabricated by employing submerged arc welding, with a temporary ceramic backing strip on the bottom side. The backing strip is removed after welding, and butt welds are inspected by ultrasonic inspection to ensure welding quality.

### 2.2. Microstructural Characteristics

**Figure 2:**
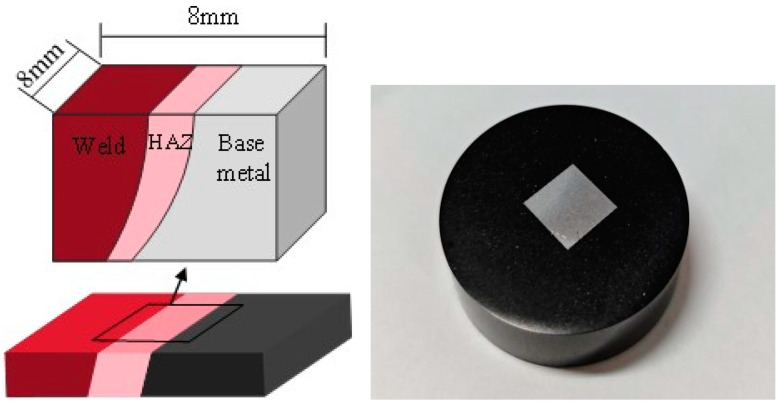
The optical microscope scan specimen and the construction.

Since the steel microstructure has a direct influence on crack propagation, the microstructure scan is first applied to the butt weld by using the optical microscope (OM) to gain comprehensive insight into different material regions. As indicated in Figure 2, the 8 mm wide square block including the weld, HAZ, and base metal regions is wire cut from the joint. The metallographic sample is then treated with polishing and etched with 4% nitric acid and alcohol corrosion. Subsequently, the metallographic analysis is performed after the washing and drying processes.

**Figure 3:**
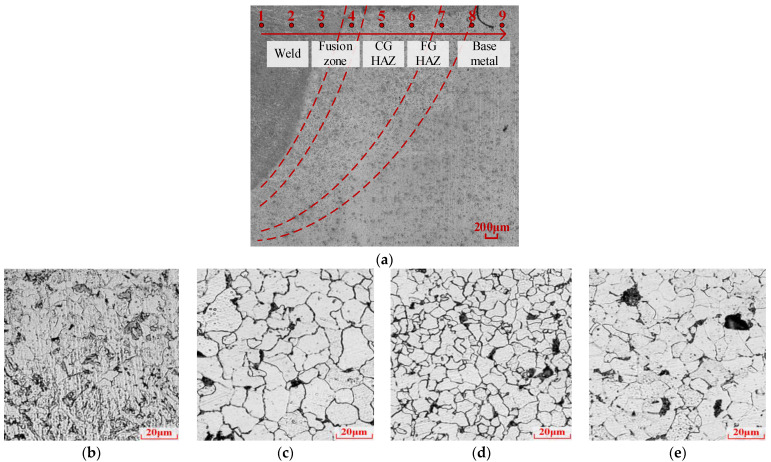
Metallographic structure: (**a**) weld joint region × 50 (Red spots indicate hardness measuring locations); (**b**) weld × 500; (**c**) CGHAZ × 500; (**d**) FGHAZ × 500; (**e**) base metal × 500.

Figure 3 gives the metallographic structure of the weld, along with the 500-time scaled scan of specific regions including the weld, base metal, and HAZ. The base steel mainly consists of ferrite (bright) grains and a small amount of pearlite grains (dark spots). The metal grains are large in size, but grain boundaries are not that clear. The equivalent size of ferrite and pearlite grains vary between 10 and 40 μm and between 5 and 20 μm, respectively. The weld has mainly pin plate-shaped ferrite, pearlite, and bainite grains with small grain sizes. Two different microstructures are observed in the HAZ. Scan spots close to the weld present relatively coarse ferrite grains (grain size around 15–30 μm) with clear grain boundaries and distributed small pearlite spots (Figure 3c) when compared to the base metal. Some HAZ scan spots close to the base metal present similar grain sizes for ferrite grains (grain size around 5–15 μm in Figure 3d) but smaller and clearer boundaries. In another parallel metallographic scan of welded Q370qENH joints, more detailed microstructure differences in HAZs are presented. There are coarse-grain HAZs (CGHAZs) and fine-grain HAZs (FGHAZs) which correspond to peak temperature regions of around 1100 °C and 900 °C, respectively, during welding [29]. Similar observations can be indicated in Figure 3a. But the boundary between the CGHAZ and FGHAZ is not clear.

### 2.3. Hardness Measurements

**Figure 4:**
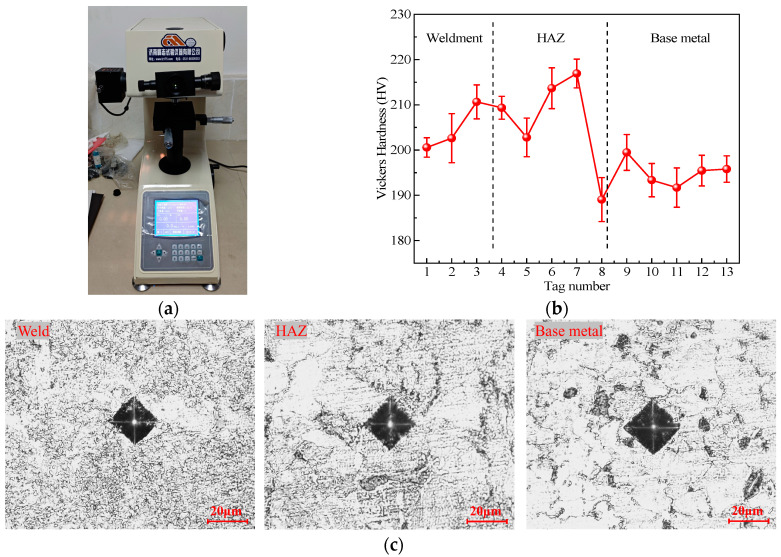
Metallographic structure: (**a**) HV-1000Z Vickers indenter; (**b**) microhardness results; (**c**) Vickers indenter imprint.

Previous references [6,7] indicate that the fatigue performance is partially related to the metal hardness. Hence, in the test, the microhardness in different weld regions is measured with the HV-1000Z Vickers indenter (Jinan, China. Figure 4a), based on GB/T 2654-2008 [30]. The testing load is 0.1 kg, and the constant load duration is 15 s. The diagonal length of the surface indentation is measured to indicate the Vickers hardness. As in Figure 3a, the measuring spots spread from the weld center to the base metal region with a distance of around 0.5 mm.

The hardness results are shown in Figure 4b. Examples of the Vickers indenter imprint in relation to the metal grains are present in Figure 4c. The imprint range is larger than grain sizes in the weld and HAZ and is much larger than the pearlite grains in base metal. Therefore, the hardness measurements reflect the comprehensive material behavior of measured regions. To avoid microhardness distractions from regional grain structures, each weld region is measured 4 times. The average value is obtained as the test hardness.

The weld spots exhibit a hardness ranging from 200.6 HV to 210.7 HV, with an average hardness of 204.6 HV. In the HAZ region, the hardness ranges between 189.1 HV and 216.9 HV, with an average of 206.4 HV. Comparatively, the base metal demonstrates relatively low hardness, ranging from 191.7 HV to 199.5 HV, with an average of 195.2 HV. The average hardness across the three regions ranks as weld > HAZ > base metal, while the extent of hardness unevenness ranks as HAZ > weld > base metal. These hardness properties align with the observed metallographic structures, in which the presence of spiculate small ferrite grains corresponds to higher metal hardness. The large-grain blur-boundary microstructure features low hardness. The HAZ has different regions of microstructures and then the hardness presents an evident variation, with the maximum and minimum hardness all presented within the HAZ region.

### 2.4. CT Specimen Design

**Figure 5:**
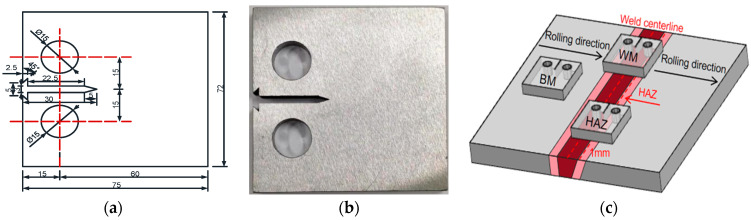
CT specimen design: (**a**) specimen dimensions; (**b**) specimen figure; (**c**) specimen locations.

Considering the weld, base steel, and different HAZ regions, a total of 46 CT specimens are designed, which can be sorted into four groups. The CT specimens are designed and fabricated according to the ASTM E647. Figure 5a illustrates the geometric details of the compact tensile (CT) specimen adopted in the fatigue crack growth (FCG) test. According to the ASTM E647 [31], all CT specimens are machined by wire-electrode cutting, and the notch direction of the specimen is right to the rolling direction of the steel plate, to simulate the general working conditions of butt welds. Figure 5c shows the CT specimen locations of base steel (B group), weld (W group), and HAZ (HAZ group) in the welded plate. Detailed positions of different group specimens in relation to the weld are also indicated. The HAZ specimen is taken with the notch centerline being 1 mm away from the weld margin (with a 6 mm distance from the weld centerline). Considering the inclined groove form, the notch in a HAZ specimen is in fact crossing the CGHAZ, the FGHAZ, and the base metal region.

To better indicate the influence of different grain sizes and microstructures in the HAZ, an additional group with CT specimens fabricated from heat-treated steels (HT group) is included. The heating process simulates temperature histories at the CGHAZ and FGHAZ. To simulate actual welding conditions, the furnace is preheated to the desired peak temperature, and then the Q370qENH strip plates are subsequently placed into the furnace. The furnace is reheated to peak temperature, and the plates are kept under the peak temperature for around 10–15 s. The steel strips are then taken out and placed on a large steel plate for air cooling. Two peak temperatures of 1100 °C and 900 °C are adopted to simulate the thermal conditions of the CGHAZ and FGHAZ, respectively. The CT specimens are fabricated from the strip plate with the same dimensions in Figure 5a. Three plate thicknesses of 6 mm, 8 mm, and 10 mm, and three stress ratios of 0.1, 0.2, and 0.5, are designed to investigate their influences on FCG properties. Due to different strength levels of the base metal, weld, and HAZ, appropriate maximum loads *P*_max_ are determined according to the preliminary tensile test results. The stress ratio indicates the ratio of the applied minimum load versus the maximum load, *P*_min_/*P*_max_. Critical parameters of fatigue test groups and specimens are tabulated in Table 5. For each case, three specimens are tested, and the two most consistent results are employed to reduce testing errors.

### 2.5. Fatigue Test Procedure

**Figure 6:**
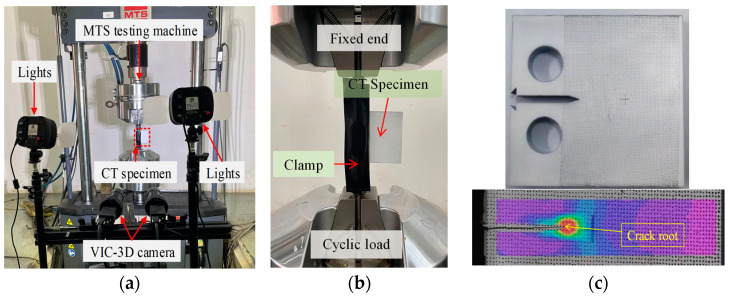
Fatigue crack growth test setup: (**a**) test setup; (**b**) specimen setup; (**c**) DIC measurement.

The fatigue tests were conducted using the MTS250 electro-hydraulic servo testing machine (Eden Prairie, MN, USA. Figure 6). The crack length is measured by the direct observation method [25,32] through a 2000-scale optical microscope (with a precision of 0.001 mm) installed at one side of the CT specimen. The other side of the CT specimen is monitored in real time with the DIC high-definition camera (Beijing, China) to identify the real-time location of the strain concentration at the crack root (Figure 6c). The crack lengths recorded by two different methods are used to verify the data effectiveness, and the average value is taken as the determined length. The fatigue test procedure is conducted based on the metallic material–fatigue testing–fatigue crack growth method GB/T 6398-2017 [33]. In order to eliminate the effect of manufacturing defects on the crack growth rate, the 5 mm precrack is produced before the formal testing, for which the length is not included in the crack length recording. The loading frequency is 10 Hz and the crack growth length of every 5000 cycles is recorded until reaching the desired crack length limit of 35 mm.

With the crack growth length *a* and corresponding loading cycle *N*, the stress intensity factor (SIF) amplitude Δ*K* can be calculated as follows:

(1) $$\Delta K=\frac{\Delta P}{B\sqrt{W}}(2+\frac{a}{W})(\frac{0.866+4.64(\frac{a}{W})-13.32{\left(\frac{a}{W}\right)}^{2}+14.72{\left(\frac{a}{W}\right)}^{3}-5.6{\left(\frac{a}{W}\right)}^{4})}{{\left(1-\frac{a}{W}\right)}^{\left(3/2\right)}}$$

where *a* denotes the crack size, and *B* and *W* indicate the specimen thickness and width, respectively.

The crack growth rate d*a*/d*N* can be obtained using the seven-consecutive-point polynomial increment method. For any selected test data point *i*, three preceding and three subsequent points are incorporated, and the quadratic polynomial is used for the seven data fitting cases, as illustrated in Equation (2):

(2) $$\overset{\wedge }{a_{i}}=b_{0}+b_{1}\left(\frac{N_{i}-C_{1}}{C_{2}}\right)+b_{2}{\left(\frac{N_{i}-C_{1}}{C_{2}}\right)}^{2}$$

(3) $$\begin{array}{c} -1\leq \frac{N_{i}-C_{1}}{C_{2}}\leq +1\\ C_{1}=\frac{1}{2}\left(N_{i-n}+N_{i+n}\right)\\ C_{2}=\frac{1}{2}\left(N_{i+n}-N_{i-n}\right)\\ a_{i-n}\leq a\leq a_{i=n},n=1,2,3 \end{array}$$

where $\hat{a_{i}}$ is the fitted value; *b*_0_, *b*_1_, and *b*_2_ are the regression parameters; and *N_i_* is the fatigue cycle of selected data point *i*. The d*a*/d*N* at *N_i_* can then be calculated by the derivative in Equation (2).

(4) $${\left(\frac{da}{dN}\right)}_{\overset{\wedge }{a_{i}}}=\frac{b_{1}}{C_{2}}+\frac{2b_{2}(N_{i}-C_{1})}{C_{2}^{2}}$$

The Paris law is often used to quantitatively describe the relationship between the FCG rate d*a*/d*N* and the SIF amplitude Δ*K* during the stable crack growth stage.

(5) $$\frac{da}{dN}=C{\left(\Delta K\right)}^{m}$$

Here, *C* is the Paris law coefficient and *m* is the Paris law exponent.

## 3. Fatigue Crack Growth Rate Results and Discussion

### 3.1. Effect of Stress Ratio

**Figure 7:**
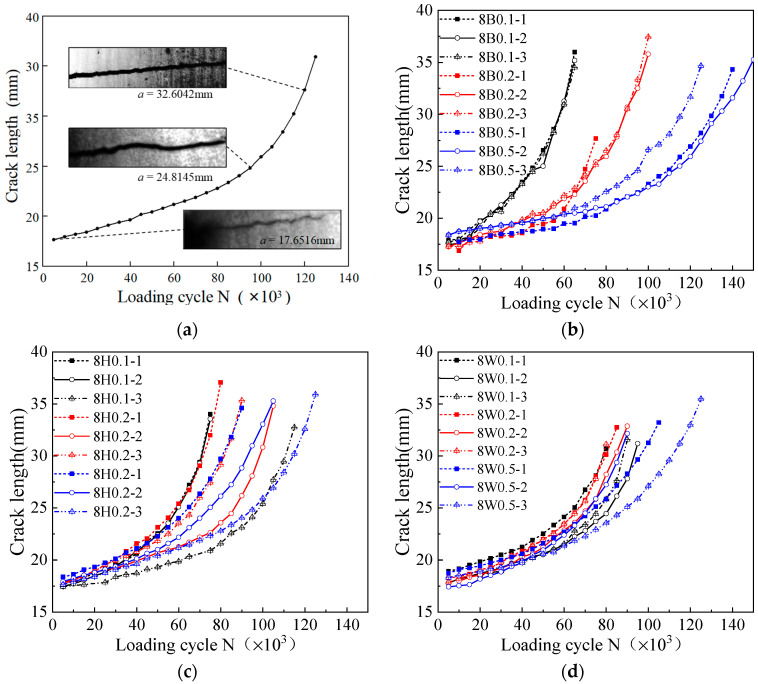
Fatigue crack growth curves: (**a**) crack propagation trajectory; (**b**) crack lengths of base metal; (**c**) crack lengths of HAZ; (**d**) crack lengths of weld.

Figure 7a illustrates the fatigue crack propagation trajectory of specimen 8HAZ0.5-3, with the corresponding *a*-*N* curve plotted alongside. The crack propagation path presents slight fluctuations, and the crack tip is sometimes hard to identify by visual observation. The incorporation of the DIC strain tracing technique effectively helps the crack length identification. Obtained crack length versus applied cycle (*N*) relations are plotted in Figure 7b–d, with the FCG data during the precrack stage and the fast initial crack propagation stage being excluded. The obtained crack length increases exponentially with an increase in the loading cycle. The 0.5 stress ratio cases generally present slow crack length developments when compared to small stress ratio cases. The increased stress ratio indicates reduced load variations during the fatigue loading, which then induces slow crack length growth under the same amount of loading cycles. Three base steel specimens of the same testing procedure present similar crack length variations. But for the HAZ and weld specimens, the crack length developments show an increased level of distraction between the three repeated specimens. The crack length propagation is different between 0.1 and 0.2 ratio conditions for the base metal, but the differences are reduced as the notch location shifts to the HAZ and to the welds. Since the crack lengths are influenced by many aspects like the precrack length and crack growth stage determinations, analyses of the crack growth rate are important.

**Figure 8:**
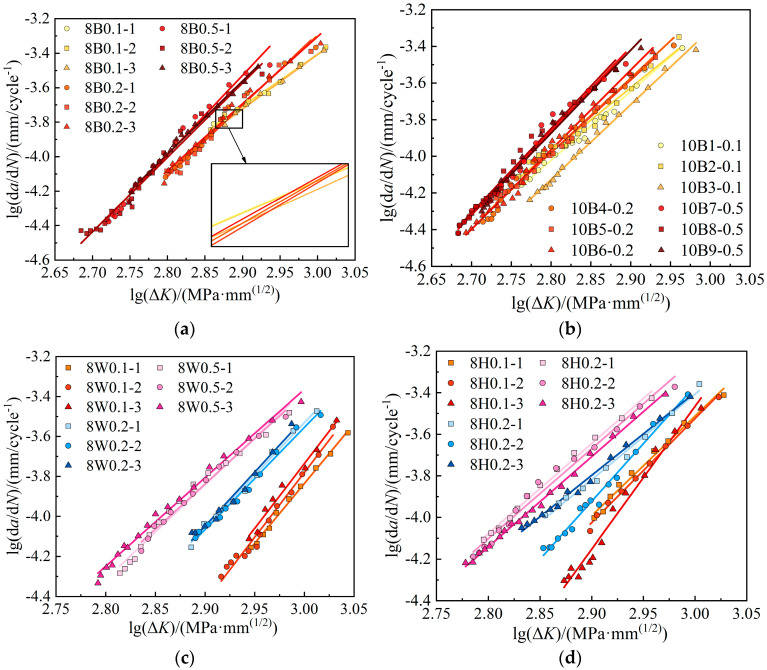
Comparison of FCG rates under different stress ratios: (**a**) base metal—8 mm thick; (**b**) base metal—10 mm thick; (**c**) weld—8 mm thick; (**d**) HAZ—8 mm thick.

Figure 8 compares the FCG rate versus the crack driving force Δ*K* in the log–log relation. The log(d*a*/d*N*)–log(Δ*K*) relation is approximately linear with a slope of *m* and an intercept of *C*, and these results prove the effectiveness of the Paris law in describing the FCG relations. As the tangents are positive, FCG rates accelerate with the crack growth development. As the stress ratio increases, the FCG rate curve generally shifts to the left, and the Δ*K* value at the onset of the steady growth stage presents a reduction trend. This phenomenon indicates that the stress ratio influences the initial crack expansion stage. The required stress intensity factor Δ*K* to enter the stable expansion phase decreases as the stress ratio increases. For a given Δ*K*, the FCG rates increase with the stress ratio *R*, indicating a faster crack growth rate under high stress ratios.

The stress ratio is controlled by varying the minimum stress value while maintaining the maximum stress value constant. At the same crack length, the low driving force (load gap between the maximum and minimum loads) caused by the high stress ratio hinders crack growth. An increased stress ratio facilitates faster propagation of the plastic zone at the crack tip. In cases with small stress ratios, the existing crack tends to experience closure or recovery at low load levels. As the stress ratio increases, the crack closure effect gradually weakens, leading to an increase in the driving force and a subsequent acceleration in the expansion rate. The influence of the stress ratio *R* is relatively modest in the base steel region, whereas *R* exhibits a more pronounced effect in the weld zone. Additionally, the heat-affected zone (HAZ) displays a higher sensitivity to stress ratios compared to the base steel.

### 3.2. Effect of Plate Thickness

**Figure 9:**
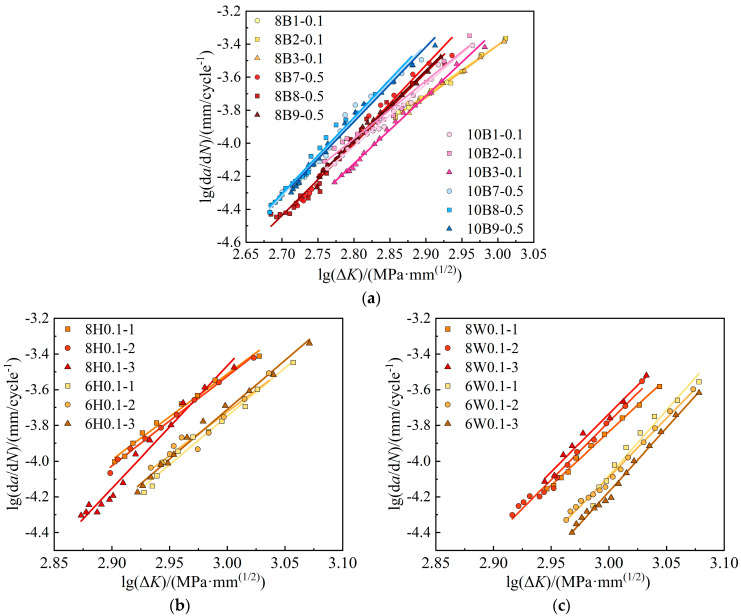
Comparison of FCG rates under different plate thicknesses: (**a**) base steel cases; (**b**) HAZ cases; (**c**) weld cases.

Figure 9 compares the FCG rates of 8 mm and 10 mm Q370qENH steel, as well as the 6 mm and 8 mm cases for the heat-affected zones and welds. In both weld and heat-affected zones, the FCG rates increase with an increase in plate thickness when under the same stress ratio. This trend is more evident in the weld. The Q370qENH base steel exhibits higher FCG rates in thick-plate specimens (10 mm) under the same stress ratio, although the differences are not distinct. These findings suggest that fracture toughness *K*_c_ tends to increase in thin plates, presenting the idea that the larger Δ*K* value is required for yielding in thin plates.

### 3.3. FCG Performance in Different Weld Regions

**Figure 10:**
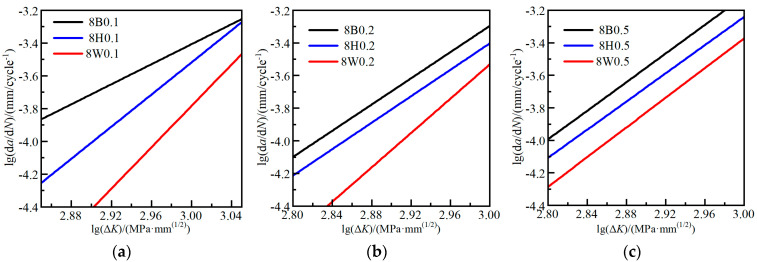
Comparison of FCG rates under different stress ratios: (**a**) 0.1 stress ratio; (**b**) 0.2 stress ratio; (**c**) 0.5 stress ratio.

Figure 10 compares the FCG rates of 8 mm CT specimens with the notch at different locations. The average FCG rate curve of each case is obtained for comparison. The results of 8H0.1-3 and 8H0.2-2, which exhibit different gradients compared to their repeat measurements as shown in Figure 8c, were excluded during the calculation. Different FCG rates are presented: The base steel presents the highest FCG rate, followed by the heat-affecting zone. The weld area exhibits the lowest rate. The FCG rate differences are most pronounced at the stress ratio of 0.1. As the stress ratio *R* increases to 0.2 and 0.5, the FCG rates of three critical locations all increase gradually, and the rate differences diminish. This phenomenon can be attributed to the crack closure effect. At the stress ratio of 0.1, there is a high level of the fatigue crack closure effect. The variances in material hardness and strength properties contribute to different degrees of crack closure at the crack tip, resulting in distinct trends in FCG rates. However, as the stress ratio increases, the crack tip closure effect weakens, and the impact of material hardness differences decreases. The FCG rates of different weld regions present similar gradients. The obtained Paris law coefficients *C* and *m* are summarized in Table 6.

**Figure 11:**
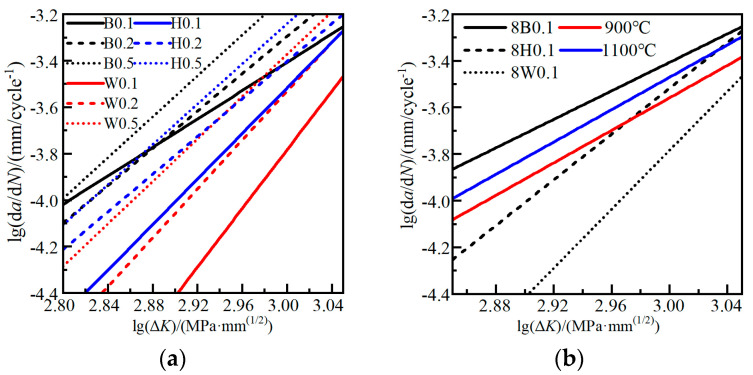
Comparison of FCG rates between different CT groups: (**a**) CT specimens from weld joint; (**b**) CT specimens from heated plates.

Since the V-shaped groove weld is used in the study, the vertical through notch in a HAZ specimen crosses different sub-regions of the HAZ regions and the base metal region. Thus, the obtained FCG rate results actually represent the overall performance of the near-weld region. Figure 11 provides a comparison of the FCG rate results between the welded joint cases and the heat-treated (HT) cases. The simulated CGHAZ1100 and FGHAZ900 cases exhibit lower FCG rates compared to the base metal cases but with similar rate gradients. Taking into account the microstructure conditions depicted in Figure 3, it can be observed that the presence of fine-grain microstructures hinders crack growth, resulting in slower FCG rate performances.

### 3.4. FCG Performance Comparison with Different Grade Steels

**Figure 12:**
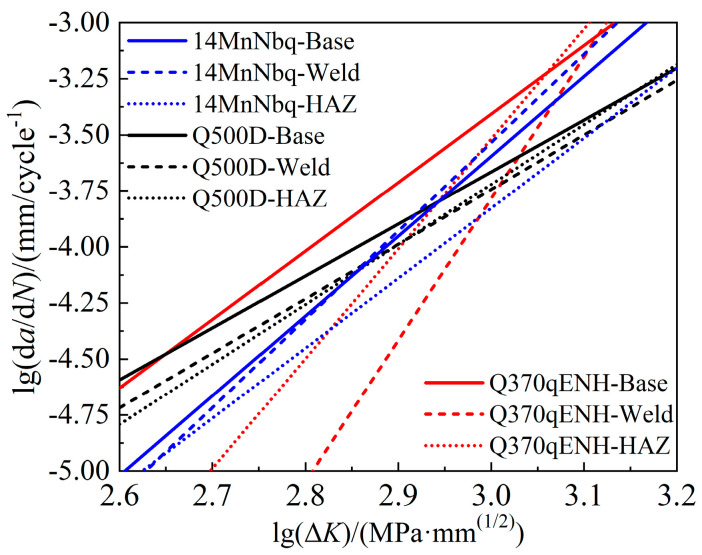
FCG performance comparisons between Q370qENH, 14MNNbq, and Q500D.

Figure 12 compares FCG rate curves of base steel, weld, and the HAZ between tested Q370qENH-, 14MNNbq-(nominal yield strength of 390 MPa) [34], and Q500D-grade steels [35] reported in the references. The three types of steels all presented slower FCG levels but higher tangents in the weld and HAZ than in the base steels. As the strength of the grades increases, the FCG rate exhibits a decreasing trend with reduced FCG levels and decreased tangents. As indicated in Figure 3, the decrease in FCG rates mainly comes from the refined grain sizes and clear boundaries in the microstructure. The high-strength steels generally possess refined steel grains and fewer impurities. Thus, the influence of microstructure tends to decrease as the steel strength grades increase. Therefore, in Figure 12, differences between base steels, weld, and HAZ regions decrease from Q370qENH to 14MNNbq and further to Q500D.

## 4. Fracture Morphology Results and Discussion

### 4.1. Fracture Surface of Fatigue Crack

**Figure 13:**
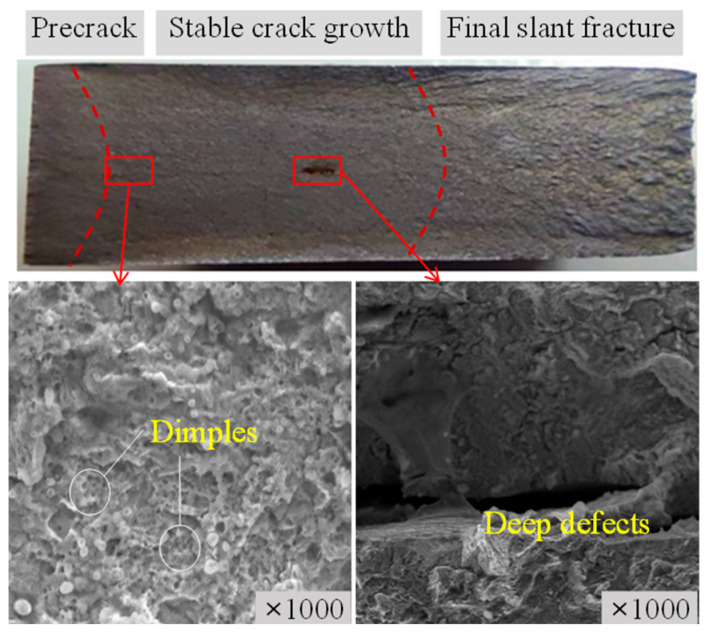
Fracture morphology of 8H0.2-2.

Fracture morphology is critical evidence for identifying crack growth characteristics in materials. FCG rates of 8H0.1-3 and 8H0.2-2 in Figure 8d present distracted log(d*a*/d*N*)–log(∆*K*) relations, specifically presenting the increased tangent *m* than corresponding repeated ones. The fracture surfaces are observed and surface scans of 8H0.2-2 are presented in Figure 13. The fracture surface corresponds to the precrack region, and the stable crack propagation region is dominated by a flat fatigue fracture surface. The rapid crack growth region features rough surfaces and tearing ridges. Abnormal FCG rate results are attributed to impurities, defects, and hidden cracks at the stable crack growth region, causing increased FCG rate-developing speeds. Microscopic morphologies of the fracture surfaces were examined via a scanning electron microscope (SEM). The microscopic morphology in the precrack region and the early crack growth region presents many dimples, indicating the microporous aggregation type fracture. The different microstructures lead to small FCG rate levels under small Δ*K* periods.

**Figure 14:**
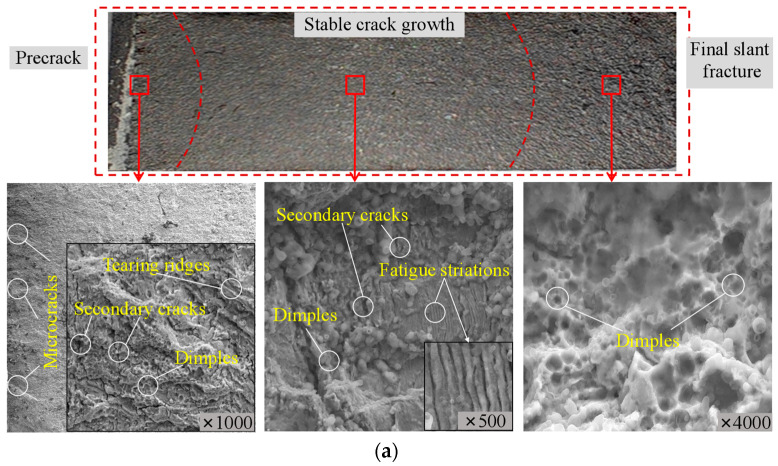

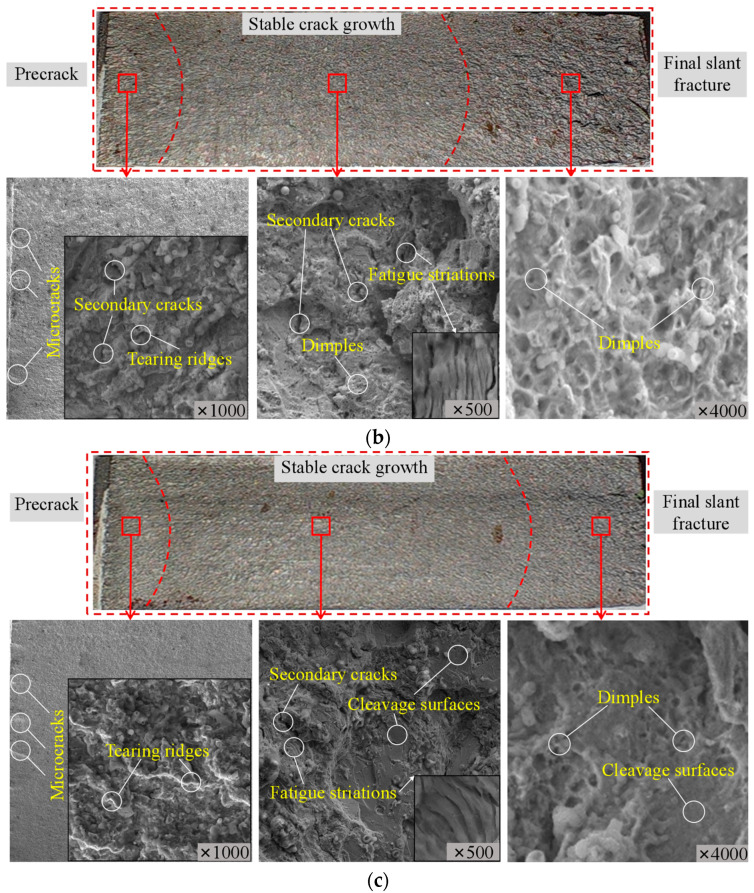
Fracture surface and microscope morphology of critical regions: (**a**) weld—8W0.1; (**b**) HAZ—8H0.1; (**c**) base steel—8B0.1.

Figure 14 compares the fracture morphologies of representative base metal, weld, and HAZ specimens. The microscope morphology of different crack-developing stages is scanned under an SEM. The boundaries between different crack-developing stages are curved lines. The precrack regions of the weld, HAZ, and base metal exhibit similar fracture morphology, and fatigue cracks primarily initiate from the notch tip. Fine cracks continue to propagate, causing numerous fine cracks resembling “river-like” patterns. In low stress ratio conditions, quasi-cleavage fractures, with cracks generally propagating along slip bands within the grains, are predominant.

Comparatively, the fracture surface of the base metal exhibits a relatively low roughness surface, while the HAZ and weld regions possess some tearing ridges, featuring rough fracture surfaces. These tearing ridges are formed under the stress concentration at the crack tip and tend to be parallel to the main crack growth direction. The presence of needle-like ferrite in the weld introduces direction variations in crack propagation, which aids in reducing the effective driving force for crack growth and consequently slowing down the fatigue crack propagation rate. In the heat-affected zone and weld regions, there are also secondary cracks and small ductile dimples present, representing the ductile deformation modes.

In the steady crack growth stage, three metal regions all present fatigue striations that form from continuous passivating and sharpening behaviors at the crack tip. The fracture mode in this stage is a combination mode of ductile fracture and fatigue striation fracture. The fatigue striation will extend the fracture path, and consume energy at the crack tip, thus partially hindering further crack growth. Due to the variations in grain boundaries and metallic compositions, the fatigue striations show uncertainties in band directions and lengths. The fatigue striations are more prominent and uniform in the weld region, followed by the HAZ. The directions are more likely to the right of the crack propagation path. The base steel exhibits fewer fatigue band features compared to the weld and HAZ cases, and the band features are slightly apparent during the later crack growth region. The band width ranking between striations is as follows: weld < HAZ < base steel. The relation aligns with the fatigue crack growth rate results that wide striations indicate faster fatigue crack growth in the base steel. Moreover, a high percentage of secondary cracks can be observed in the weld and HAZ cases, while the base steel has fewer secondary cracks but a high level of cleavage fractures.

The final slant fracture stage of three weld region cases all present ductile dimple features, indicating the increasing prominence of ductile fracture mechanisms. The dimple structure uniformity is weak in the base steel. The base metal also exhibits some cleavage platforms in the final fracture region, indicating a mixture failure mode of brittle and ductile fracture. The HAZ presents an increased percentage of ductile dimples, with relatively uniform dimple sizes. The weld has refined grains, and the percentage of ductile fracture increases.

### 4.2. Fracture Surfaces Under Different Stress Ratios

**Figure 15:**
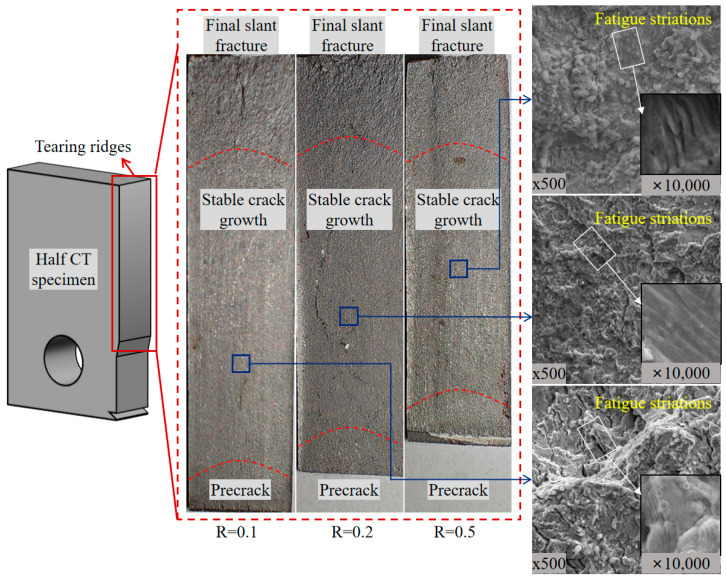
Fracture surface and microscope morphology of critical regions.

Figure 15 compares the fracture surfaces of an 8 mm thick base metal under 0.1, 0.2, and 0.5 stress ratios. The crack growth length shortens and the macroscopic roughness decreases in the stable growth region as the stress ratio increases. The fracture surface of the final fraction region is rough in high stress ratio cases. In the 0.5 stress ratio case, the crack front of the stable crack growth region exhibits an arc shape, indicating the non-uniform stress state at the crack front across the thick direction. Furthermore, the fatigue bands become widened in high stress ratio cases, indicating the increased FCG rate. These results are consistent with the FCG rate variations.

### 4.3. Fracture Surfaces Under Different Plate Thicknesses

**Figure 16:**
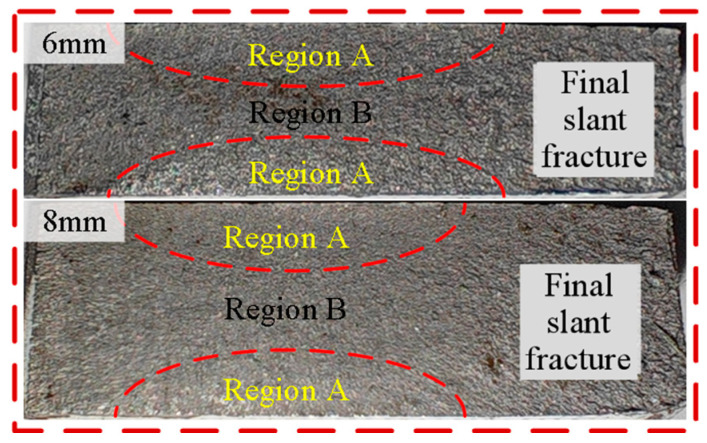
Fracture surface of 6 mm and 8 mm thick 0.1 stress ratio cases.

The plate thickness primarily influences the stress state across the fracture surfaces. As indicated in Figure 16, region A mainly experiences the plain stress state. The center region B experiences the transition between the plain strain state to the plain stress state, presenting as slight arch transition ridges in the fracture surfaces as the dash boundary lines in Figure 16. The domination and enlarged area of region A indicate the widened plastic deforming region, which then leads to slow crack growth in the specimen.

## 5. Conclusions

A comprehensive study on the microstructural properties and fracture crack growth behaviors was performed on the Q370qENH bridge steel butt weld joints. A total of 46 compact tensile specimens of base steel, butt weld, and HAZ regions were tested. Different plate thicknesses and stress ratios were considered. The fracture surface observations were performed after fatigue tests. The Paris law coefficients of different regions of Q370qENH welds were presented. By analyzing the test data, the following conclusions were obtained:

1. The microstructure of the Q370qENH bridge steel is mainly large-grain ferrite with nonclear grain boundaries. The weld is predominated with pin plate ferrite, pearlite, and bainite with small grain sizes. Compared to the base steel, the HAZ has a smaller size but clearer boundary ferrite grains with small distributed pearlite grains. The average hardness across the butt weld is decreasingly ranked as follows: weld > HAZ > base metal.
2. The stress ratio has a significant influence on the FCG behavior of Q370qENH joints, but the influencing extent is different for different regions. The larger the stress ratio, the faster crack growth is presented, and the Δ*K* value at the onset of the steady growth stage tends to decrease. The effect of the stress ratio is prominent in the weld, and the influencing extent decreases in the HAZ and is modest in the base steel. The FCG rate of Q370qENH steel increases with plate thickness under the same stress ratio. The differences induced by plate thickness are more evident in the HAZ and weld specimens.
3. The Q370qENH base steel presents the highest FCG rate, followed by the HAZ, while the weld exhibits the lowest rate. The simulated HAZ cases (CGHAZ1100 and FGHAZ900) also present slower FCG than the base steel. The heating and cooling processes during welding result in improved microstructures with refined grain sizes and fewer impurities in the Q370qENH steel, thus leading to improved FCG performance in the HAZ and weld regions.
4. The fracture mode in the steady crack growth stage is a combination of ductile fracture and fatigue striation fracture. The fatigue striations are more prominent and uniform in the weld region, followed by the HAZ. The crack growth length shortens and the macroscopic roughness decreases in the stable growth region as the stress ratio increases.

## Figures and Tables

**Table 1:** Chemical composition of bridge weathering steel Q370qEN from supplier (% weight).

Content	C	Si	Mn	P	S	Ni	Cr	Cu	V	Ti
(wt%)	0.094	0.27	1.37	0.016	0.002	0.32	0.44	0.026	0.0066	0.016
GB/T 714	≤0.1	0.15–0.5	1.1–1.5	≤0.020	≤0.005	0.3–0.4	0.4–0.7	0.25–0.3	0.01–0.1	0.03–0.06

**Table 2:** Chemical composition of soldering flux SJ105N (% weight).

Content	CaO + MgO	Al_2_O_3_ + MnO	SiO_2_ + TiO_2_	CaF_2_	P	S
Guarantee value	26~29	28~35	15~20	18~21	≤0.030	≤0.030

**Table 3:** Chemical composition of welding wire TH500-NQ-III (% weight).

Content	C	Si	Mn	P	S	Cu	Cr	Ni
Guarantee value	≤0.12	≤0.35	1.00~1.60	≤0.025	≤0.015	0.26~0.60	0.30~0.90	0.40~0.80

**Table 4:** Mechanical properties of Q370qENH specimen steel.

Yield Strength *f*_y_	Ultimate Strength *f*_u_	Elongation	*V* Impact Energy/−40 °C
386 MPa	580 MPa	22.8%	82 J

**Table 5:** Specimen parameters of Q370qENH steel.

Testing Group	Thickness /mm	No.	Stress Ratio	*P*_max_	Testing Group	Thickness /mm	No.	Stress Ratio	*P*_max_
Base metal (B)	8	8B0.1	0.1	8.5 kN	Heat-treated (HT)	8	CGHAZ1100	0.1	8.5 kN
		8B0.2	0.2						
		8B0.5	0.5						
	10	10B0.1	0.1				FGHAZ900	0.1	
		10B0.2	0.2						
		10B0.5	0.5						
Weld (W)	6	6W0.1	0.1	12 kN	Heat-affected zone (HAZ)	6	6HAZ0.1	0.1	10 kN
	8	8W0.1	0.1			8	8 HAZ0.1	0.1	
		8W0.2	0.2				8 HAZ0.2	0.2	
		8W0.5	0.5				8HAZ0.5	0.5	

**Table 6:** Obtained Paris law coefficients.

Testing Group	No.	*m*	log*C*	Testing Group	No.	*m*	log*C*
Base metal (B)	8B0.1	3.0568	−12.5774	Heat-treated (HT)	CGHAZ1100	3.4700	−13.8810
	8B0.2	4.0134	−15.3362				
	8B0.5	4.3972	−16.3055				
	10B0.1	3.7870	−14.6457		FGHAZ900	3.4861	−14.0175
	10B0.2	4.1276	−15.5323				
	10B0.5	4.5688	−16.6537				
Weld (W)	6W0.1	7.0039	−25.1250	Heat-affected zone (HAZ)	6HAZ0.1	5.2682	−19.5326
	8W0.1	6.3169	−22.7348		8 HAZ0.1	4.9029	−18.2270
	8W0.2	5.2573	−19.3037		8 HAZ0.2	4.0501	−15.5531
	8W0.5	4.5623	−17.0595		8HAZ0.5	4.3312	−16.2344

## Data Availability

The original contributions presented in the study are included in the article, further inquiries can be directed to the corresponding author.
